# A Fully Adapted Headstage With Custom Electrode Arrays Designed for Electrophysiological Experiments

**DOI:** 10.3389/fnins.2021.691788

**Published:** 2022-03-03

**Authors:** Flávio Afonso Gonçalves Mourão, Leonardo de Oliveira Guarnieri, Paulo Aparecido Amaral Júnior, Vinícius Rezende Carvalho, Eduardo Mazoni Andrade Marçal Mendes, Márcio Flávio Dutra Moraes

**Affiliations:** ^1^Programa de Pós Graduação em Neurociências, Núcleo de Neurociências, Departamento de Fisiologia e Biofísica, Instituto de Ciências Biológicas (ICB), Universidade Federal de Minas Gerais (UFMG), Belo Horizonte, Brazil; ^2^Programa de Pós-Graduação em Engenharia Elétrica, Departamento de Engenharia Eletrônica (DELT), Escola de Engenharia, Universidade Federal de Minas Gerais (UFMG), Belo Horizonte, Brazil

**Keywords:** extracellular electrophysiology recording, laboratory equipment, Intan Technologies, electrode arrays, tungsten wires

## Abstract

Electrophysiological recordings lead amongst the techniques that aim to investigate the dynamics of neural activity sampled from large neural ensembles. However, the financial costs associated with the state-of-the-art technology used to manufacture probes and multi-channel recording systems make these experiments virtually inaccessible to small laboratories, especially if located in developing countries. Here, we describe a new method for implanting several tungsten electrode arrays, widely distributed over the brain. Moreover, we designed a headstage system, using the Intan^®^ RHD2000 chipset, associated with a connector (replacing the expensive commercial Omnetics connector), that allows the usage of disposable and inexpensive cranial implants. Our results showed high-quality multichannel recording in freely moving animals (detecting local field, evoked responses and unit activities) and robust mechanical connections ensuring long-term continuous recordings. Our project represents an open source and inexpensive alternative to develop customized extracellular records from multiple brain regions.

## Introduction

In 1957, the Nobel laureates David Hunter Hubel and Torsten Nils Wiesel developed sharpened insulated tungsten microelectrodes to record the extracellular action potentials in the primary visual cortex of anesthetized and unrestrained cats (Hubel, 1957; Hubel and Wiesel, 1962). Beside the scientific breakthrough regarding visual system physiology (i.e., the complex cortical representation of visual information), the tungsten microelectrodes, with tip diameter less than a micron, formed the basis for the development of modern neural probes (Szostak et al., 2017; Hong and Lieber, 2019).

Since Hubel and Wiesel’s discovery, electrophysiologists have bolstered creativity-guided hypotheses to design specialized electrode arrays aimed at recording not just a small number of single units but several neuronal populations at once (Szostak et al., 2017; Hong and Lieber, 2019). By making use of metal (Dowben and Rose, 1953; Hubel, 1957; Green, 1958; McNaughton et al., 1983) or silicon (Jones et al., 1992; Rousche and Normann, 1998; Buzsáki et al., 2015; Jun et al., 2017) a wide range of electrodes with different sizes, shapes, and geometries, has been used to sample brain activity. Moreover, alongside the development of specialized electrodes, electrophysiology data acquisition systems have reached a high degree of accuracy, miniaturization and data processing capabilities that allow recording from several channels simultaneously. Nowadays, with the low noise integrated amplifier chips, that digitize signals on the spot (e.g., Intan Technologies; Harrison, 2007), researchers are able to record thousands of neurons in a single multi-channel probe (Jun et al., 2017) while transmitting the digitized data from each channel through a very small number of wires.

However, the main problem of these state-of-the-art tools is the high costs that make it impossible to perform cutting-edge experiments, especially in newly formed under budget laboratories, or in developing countries with a lack of financial resources (Hallal, 2021). Furthermore, in the case of manufactured electrode arrays, the high costs are associated with manufacturer limitations such as the number of brain substrates that can be recorded simultaneously, the headstage sizes, and the need for special connectors and cables that drive up costs even more (Voigts et al., 2013; Jun et al., 2017; Chung et al., 2019).

To successfully deal with the costs and in an attempt to create more versatile electrode arrays, some authors have developed creative and custom-made solutions for recording the Local Field Potential (LFP) and the multiunit activity in freely moving animals (Polo-Castillo et al., 2019; França et al., 2020). Nevertheless, these solutions still need expensive commercial connectors, which are not easily recoverable after the experiment, present a reasonably rigid geometry that does not allow recording widely distributed brain regions and often have mechanical problems if long-term continuous recordings are intended, such as small fractures in their contacts during repeated connections and disconnections, leading to lost connectivity or permanent damage.

In this work, we describe a full low-cost headstage for electrophysiology experiments with scalable electrode arrays, custom-made to the experimental design. In other words, we present a simple and new architecture for electrode placement, so that a different number of electrode bundles can be widely distributed over the brain. In addition, in order to provide more affordable costs, we propose an adaptation to the RHD2000 headstage system (Intan Technologies^®^), associated with a different connector, to replace the commercial costly ones commonly used in the scientific community (Omnetics Connector Corporation).

## Materials and Methods

### Overview

Considering that the main objective of this work is to develop an inexpensive tool to perform electrophysiology recordings, we designed our own printed circuit boards and purchase materials from bulk suppliers, except the integrated amplifier chip developed by Intan Technologies (Harrison, 2007) and the data acquisition system from Open Ephys (Siegle et al., 2017). Our design is fully compatible with Open Ephys open-source solution available to the scientific community. It is worth mentioning that the Intan chip performs the most demanding tasks of data acquisition right at the recording site, that is, it amplifies, multiplexes, digitizes each channel with 16-bit resolution, and measures impedance (Harrison, 2007).

**FIGURE 1:**
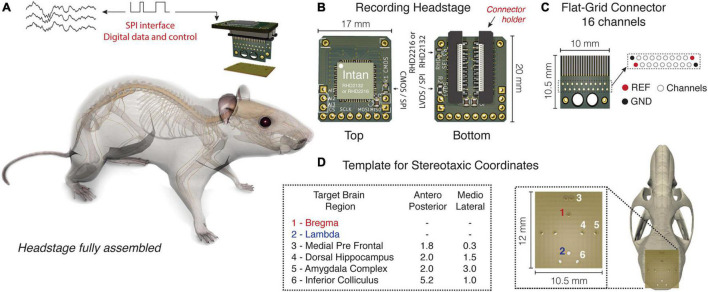
Headstage adapted from the Intan RHD headstage system with a flexible printed circuit (SMD/FPC) connector. **(A)** Headstage fully assembled in real size. **(B)** Top and bottom view of the headstage. 2132 or 2216 Intan chips can be used according to the circuit configuration. The arrow highlights the black lock on the bottom that holds the headstage to the Flat-Grid Connector. **(C)** Flat-Grid connector board with sixteen channels, reference (REF) and ground (GND), designed to connect to the SMD/FPC connector. **(D)** Fiberglass board punctured with small holes according to the chosen stereotaxic coordinates. A variable number of electrodes can be aligned and fixed from distinct dorsoventral configurations.

Our solution is composed by three main parts: (1) headstage with RHD2000 chipset and a SMD/FPC (Flexible Printed Circuit) female connector; (2) a SMD/FPC compatible flat shaped printed circuit board (to be implanted during surgery) with a grid of through holes for soldering electrodes; (3) a fiberglass board with drilled stereotaxic coordinates where the electrodes are aligned and fixed (Figure 1). Moreover, extra adapters were developed to convert Omnetics to the SMD/FPC connector (in order to allow the use of previous commercial solutions), and generic tools were made via 3D printing to facilitate the manufacture and handling of the electrode arrays (Supplementary Material).^1^

### Recording Headstage

The recording headstage, although essentially similar to the Intan Technologies standard, has five important changes: (1) Based on some solutions already commercially developed (Low-profile SPI Headstage 64ch)^2^ the circuit was built in a horizontal setup, significantly reducing damage due to excessive torque induced by sudden movements, long term continuous recordings or seizures. In addition, the lower torque imposed by the horizontal setup may facilitate animal movement during the behavioral tasks; (2) With a short circuit in the “R3” pad, or just left empty, RHD 2216 chip (16-channel with differential inputs) or RHD 2132 chip (32-channel with unipolar inputs and common reference) can be, respectively, chosen. In other words, the R3 pad short circuits the reference electrode to the RHD2216 ground (pin-10), thus allowing the differential (bipolar) amplifier inputs; (3) With a short circuit in the “R1” pad and “R2” pad empty, or a short circuit in the “R2” pad and “R1” pad empty, the two types of SPI communication, CMOS or low voltage differential signaling (LVDS) can be, respectively, chosen; “R1” and “R2” pads are connected to the LVDS_en (digital input; pin-30). According to the Intan datasheet, when LVDS_en is pulled high, a short circuit through R2 to VCC, communication with the SPI is conducted using LVDS. When LVDS_en is pulled low, a short circuit through R1 to the Ground, SPI communication uses CMOS signaling (Intan Technologies). (4) The standard Omnetics connector (i.e., model: A79042 or A79046) has been replaced with a SMD/FPC (Flexible Printed Circuits) connector (MKTechnic—model: FPC0520VH-20P. VERTICAL, TYPE 0.5MM, PITCH 2MM), which reduced costs significantly and can be easily replaced in case of damage; (5) The standard Omnetics connector (PZN-12 polarized nano connectors), whose output was intended for the SPI communication, has been exchanged for through holes (pitch—2 mm) which makes possible to solder either pin bar connectors or wires directly (Figure 1B). More information and the circuit design can be found in our repository on GitHub.^3^

### Flat-Grid Connector

The modern technology of chronic electrodes most often involves Omnetics connectors that are implanted into the animal’s skull (Royer et al., 2010; Juavinett et al., 2019). After weeks of experimental procedures, the reuse of these connectors becomes almost impossible, since they are fixed with resistant cements. When removed, they are damaged either by the procedure or by the action of the solvents commonly used to dissolve the dental resins commonly used for fixation.

To make chronic experiments more accessible we design a circuit board (10.5 mm × 10 mm × 0.4 mm) that works as a “rigid” flat cable. Basically, at the top of this small PCB there is an interface to connect to the SMD/FPC connector, in the middle there is a grid of twenty contacts (through holes with 0.2 mm each separated by 0.5 mm; two grounds, two references and sixteen contacts for sixteen channels) where the electrodes are soldered, and at the bottom there are bigger holes that, in some instance, depending on the electrode arrays arrangement, can assist the cement fixation at the skull during the implant surgery (Figure 1C), since the cement can pass through the openings covering the connector base and the skull on both sides properly. It’s important to highlight that the SMD/FPC connector features a small black lock on the bottom that strongly holds the headstage to the Flat-Grid Connector (Figures 1A,B).

### Template for Stereotaxic Coordinates

Most commercial electrodes usually have parallel distribution, that is, each shank is side by side and separated from each other by micrometers (Ulyanova et al., 2019). A similar parallel distribution has also been used by other authors who have sought to develop new and inexpensive alternatives for electrophysiological records (França et al., 2020).

In an attempt to develop a more versatile way that meets the multichannel recording of widely distributed neural networks, a fiberglass PCB was punctured with small holes. Each hole is drilled with 400 μm precision and distributed as a function of an anteroposterior and mediolateral stereotaxic coordinate (Paxinos and Franklin, 2012), where a variable number of electrodes can be aligned and fixed from distinct dorsoventral configurations (e.g., Figure 1D).

### Wires

**FIGURE 2:**
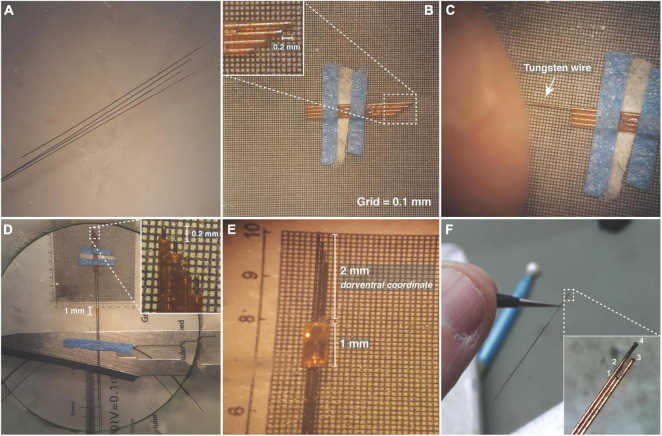
Designing electrode arrays. **(A)** Tungsten wires 50 μm in diameter. **(B)** Silica capillary tubes fixed side by side in a microscope micrometer calibration ruler, to form a matrix guide. **(C)** Tungsten wire passing through the silica tube. **(D)** Tungsten wires fixed with tips spaced by 200 μm. **(E)** 1 mm silica tube used as a sliding sleeve to define the full length of the electrode array as well as the dorsoventral coordinate. **(F)** Amplified electrode array showing the tip configuration.

In this work we choose Tungsten microwires 99.5% S-Formvar with 50 μm internal diameter (California Fine Wire Co.) for the multi-wire arrays (Nicolelis, 2007; Figure 2A). Tungsten wires are commonly used for electrophysiological records and are known for their high rigidity, durability, and high impedance (Hubel, 1957). Stainless steel with 0.127 mm internal diameter, Teflon-coated wires (Model 791400, A-M Systems Inc., Carlsborg, WA, United States) were used as reference and ground.

### Assembly Process

In order to record different brain regions and their respective neural substrates, electrode arrays with several tungsten wires each could be built and attached to the fiberglass board according to the previously established stereotaxic coordinates (e.g., Figure 1D). In turn, each wire tip from an electrode array could be spaced according to the experimental purpose (e.g., Figures 2B,E,F).

In what follows we describe one template type for stereotaxic coordinates that have been used in experiments in our laboratory. Each step of headstage manufacturing will be commented on below. The time required for the complete development of one full arrangement is around 4–5 h for experienced individuals. However, the manufacturing time depends on the number of desired electrodes and the number of target brain areas for recording. More details can be found on Supplementary Material or GitHub (see text footnote 3).

#### Step 1—Electrode Array

(1) Tungsten wires should be carefully cut to different sizes in a flat shape. We suggest using Carbide Scissors (Dr. Slick Co., Belgrade, Serbia), which has sufficient strength and does not impair the desired shape and insulation surrounding the electrode tip. Each tip needs to be checked under a microscope, so that the cut accuracy is maintained. Cutting in different sizes will facilitate their identification later (Figure 2A).
(2) For the sizing of the electrode arrays, fused silica capillary tubes 75 μm internal diameter and 155 μm external diameter (Polymicro Technologies, Arizona, United States)—were fixed side by side in a microscope micrometer calibration ruler, to form a matrix guide. This matrix was used for the correct wire alignment and to ensure a specific distance between each wire tip. This distance was in accordance with the dorsal-ventral stereotaxic coordinates of the chosen brain substrates (Figure 2B).
(3) Each tungsten wire should pass in isolation in each silica tube. Do not use tweezers or any tool that could compromise the wire insulation (Figure 2C).
(4) A fused silica tube with 1 mm length, 200 μm internal diameter, and 350 μm external diameter should hold all wires on the rear surface. The wires, in turn, must be fixed to a removable surface (Figure 2D).
(5) The 1 mm silica tube should be used as a sliding sleeve that, positioned on the rule, will define the full length of the electrode array. This length is related to the dorventral coordinate regarding the cerebral region intended to record. It is important to highlight that the dorventral coordinate through the brain is doubly confirmed through the stereotaxic frame during the surgery. After the desired positioning, a small drop of super glue (TEK BOND Saint-Gobain, number 793, São Paulo, Brazil) can be used to hold the wires next to the tube (Figure 2E).

#### Step 2—Fixing the Electrode Arrays to the Template

**FIGURE 3:**
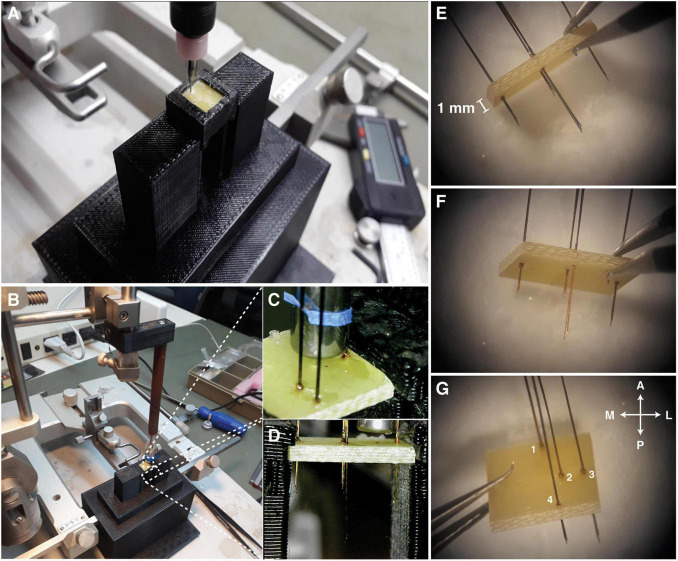
Fixing the electrodes to the template. **(A)** Support base designed to hold the fiberglass board while being drilled by a drill. **(B)** Support base attached to the stereotaxic frame. **(C)** Each electrode array must be carefully positioned at each coordinate and glued with a small drop of super glue. **(D)** Lateral view of the positioned electrode arrays. **(E–G)** Template prepared with all electrode arrays. Antero < = > Posterior (A < – > P), Medial < = > Lateral (M < = > L) views.

(1) The fiberglass board (thickness: 1 mm) needs to be punctured with small holes (PCB drill bits—0.4 mm), according to the stereotaxic coordinates chosen for the electrode array implant (Figure 3A). A support base to hold the fiberglass board was designed on a 3D printer, and this can in turn be fixed on the stereotaxic frame (Figures 3A,B and Supplementary Video 1).^4^
(2) On the stereotaxic frame, each electrode array should be positioned in the respective coordinate previously punctured on the board (Figures 3B–G and Supplementary Video 2). After the desired positioning, a small drop of super glue can be used to hold the electrode arrays next to the board.

#### Step 3—Soldering the Electrode Arrays to the Flat-Grid Connector

**FIGURE 4:**
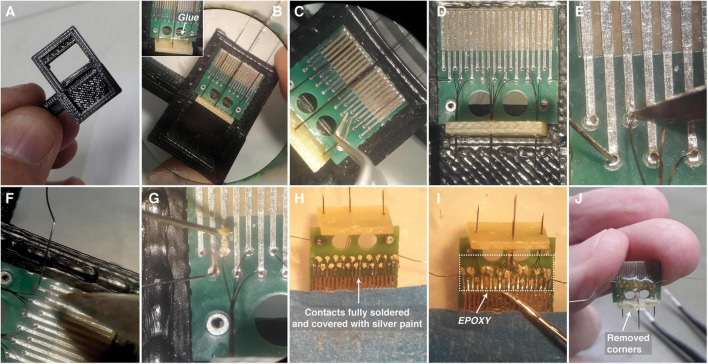
Preparing the Flat-Grid Connector. **(A,B)** Support base to hold both the flat-grid connector and the fiberglass board. The white arrow at B highlights where the glue is positioned to fix the Flat-Grid Connector to the fiberglass board. **(C,D)** Each wire needs to be inserted through the grid contacts. **(E)** The insulation of each wire needs to be removed. **(F)** Small amount of weld covering the contact between the wire and the grid. **(G)** Silver paint applied to short-circuit the contact. **(H)** Electrode arrays and Flat-Grid Connector at the posterior and bottom view fully soldered and covered with silver paint. Wires cut close to the grid. **(I)** Grid contacts covered by a thermosetting polymer (EPOXY). **(J)** Finished set with board corners removed.

(1) A small support base to hold both the flat-grid connector and the fiberglass board, with pre-prepared electrode arrays, was designed using a 3D printer. This support ensures that tungsten wires will not bend during any manipulation. In addition, the holder facilitates both soldering and fixing wires over the grid (Figure 4A; see text footnote 4).
(2) After connecting the flat-grid connector and the fiberglass board in the holder a small drop of super glue can be used to hold both (Figure 4B).
(3) With a tweezer, make the insertion of each tungsten wire through the grid contacts. Be careful with the positioning of each electrode, in the correct channel contact. Remember that each electrode has a different length and this can be used as a position marker (Figure 4C).
(4) In the same way, as in item 3, put the stainless steel wires on the reference and ground positions on the left and right sides of the grid. Note that the positioning of the ground and reference can be performed alternately (ground on the left and reference on the right or vice versa) (Figures 1C, 4D).
(5) With a sharp blade remove the insulation of each wire next to the contact on the grid (Figure 4E).
(6) Use a pointed solder iron with a small amount of weld to cover the contact between the tungsten wire and the grid (Figure 4F). Be aware that Tungsten is a refractory metal that needs special conditions to be soldered. Thus, we recommend using silver paint (SPI Supplies^®^; Pennsylvania, United States) to ensure a short-circuit between the tungsten wire and the grid contact (Figure 4G). In our experience, both methods should be used because the weld does not only fix the wire but the heat ensures the melting of the insulation.
(7) Remove the parts from the support base and do the same procedures described in items 5 and 6 on the back of the flat-grid connector. Cut the wires close to the grid (Figure 4H).
(8) Cover all grid contacts, on both sides, with a thermosetting polymer (Epoxy resin, Devcon^®^, Itw Performance Polymers, Massachusetts, United States) (Figure 4I).
(9) Optionally, the fiberglass board corners can be removed with a rotatory tool (e.g., mini cordless electric drill with a sanding band) to fit better on the animal skull (Figure 4J).

#### Step 4—Electroplating

In order to reduce the impedance imposed by the small tungsten wire diameter and possible differences in the surrounding tips insulation each tip was electroplated with gold (9V, 0.075 mA; Direct Current over 60s) in a handmade system built for this purpose.^5^ The impedance was measured in ACSF (artificial cerebrospinal fluid) solution at 25^°^C [NaCl (127 mM), KCl (2 mM), CaCl_2_ (2 mM), MgSO_4_ (2 mM), NaHCO_3_ (26 mM), KH_2_PO_4_ (1.2 mM), HEPES (13 mM); pH 7.4], by the Intan RHD2132 via Open Ephys acquisition system (Siegle et al., 2017; Table 1).

**TABLE 1:** Impedance measured by INTAN RHD2132.

Ch	1	2	3	4	5	6	7	8	9	10	11	12	13	14	15	16
Z	6.6	5.5	3.8	5.6	4.5	30.2	6.8	3.2	92.0	63.5	5.1	11.7	28.0	24.5	9.7	7.7

*Ch, channels; Z, impedance in KΩ.*

### Ethics Statement

The data and experimental procedures shown in this paper are from two male mice (C57BL/6 strain) recorded during two different experiments carried out in our laboratory.

All procedures were approved by the Institutional Animal Care and Use Committee at the Universidade Federal de Minas Gerais (CEUA-UFMG: 198/2019), conducted in accordance with Conselho Nacional de Controle de Experimentação Animal (CONCEA) guidelines defined by Arouca Act 11.794 under Brazilian federal law. CEUA directives comply with National Institutes of Health (NIH) guidelines for the care and use of animals in research.

### Surgical Procedure

Before the surgical procedure the electrode arrays were submerged in liquid enzymatic cleaner 0.5% for 5 min (Zymedet Gold 5-E—Prolink; Dental Cremer Produtos Odontológicos S.A.; Santa Catarina, Brazil), followed by rinsing with distilled water. Then it was allowed to dry in a laminar air flow cabinet under ultraviolet light (Pachane^®^, model: PA 050; São paulo, Brazil) for at least an hour.

The mice were anesthetized with Isoflurane in Oxygen at concentrations of 2–4% for induction and 0.5–2.0% for maintenance. After the absence of reflexes and signs of pain, the head surface was shaved and then the animal was positioned in a stereotaxic frame (Stoelting, Wood Dale, IL, United States). The constant flow of anesthetic was offered through a 3D printed mask designed in our laboratory. After asepsis with alcohol (70%, topical) and povidineiodine solution (7.5%, topical), local anesthesia with lidocaine clorohydrate-epinephrine [1% (wt/vol), 7 mg/kg] was applied and the scalp was removed to expose the skull.

Small holes at the top of the skull were made with a 0.5 mm drill according to stereotaxic coordinates previously defined in the template board (Figure 1D), in turn, the dorso-ventral coordinates were defined according to the length of the electrode arrays. Each array was designed with four electrodes, with tips spaced by 200 μm (Figure 2).

At the end, stainless steel screws were implanted on the contralateral side as the reference electrode (0 V) and another one as the ground and with a sharp blade the wires insulation was removed near to the screws, being properly soldered to keep contact.

**FIGURE 5:**
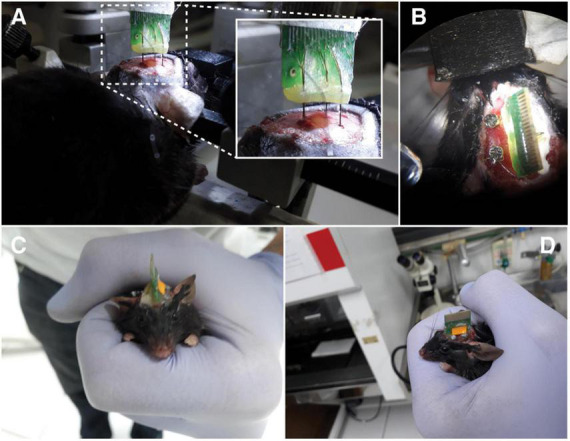
Surgical procedure. **(A)** Electrode arrays being positioned in the animal brain. **(B)** Top view. Fixing screws used as a reference and ground. **(C,D)** Animal after surgery. Anterior and lateral views, respectively.

In mouse 1, four electrode arrays were distributed as follows: Medial Pre-Frontal Cortex (mPFC), 3.0 mm; Amygdala Complex (AMY), 4.8 mm; Dorsal Hippocampus (dHPC), 2.0 mm; Inferior Colliculus (IC), 2.2 mm (Figures 1D, 5). In mouse 2, one electrode array was positioned only in the Inferior Colliculus (IC), 2.2 mm. At the end of the procedure, the implant was fixed to the skull with dental acrylic (Figure 5).

Following the surgery, animals received an intramuscular injection of penicillin-G benzathine, a broad-spectrum antibiotic cocktail (Pentabiotic, 56.7 mg/kg in a 0.1 mL volume); a subcutaneous injection of anti-inflammatory analgesic (Banamine; 0.5 mg/kg flunixin meglumine in a volume of 0.3 mL) and a subcutaneous injection of opioid analgesic (Tramadol; 20 mg/kg s.c. in a volume of 0.1 mL) and were allowed a recovery period of 7 days.

### Electrophysiological Record and Behavioral Protocol

The raw electrophysiological signals were obtained from the Intan RHD2132 via Open Ephys acquisition system. All records were sampled at 30 kHz and stored as continuous data files (*. continuous) (Siegle et al., 2017).

The animals used in the experiment were placed in a square open-field arena (30 cm × 30 cm × 30 cm) where an auditory stimuli protocol was performed. The protocol consisted of five auditory stimuli that evoked an entrainment by applying a long-lasting amplitude-modulated tone. This auditory stimulation is currently used in our laboratory for learning and memory tasks (Lockmann et al., 2017; Simões et al., 2020).

A 12-bit digital-to-analog converter at Arduino Due was used to generate the auditory stimuli (Picton et al., 2003; Amaral-Júnior et al., 2019). The algorithm behind the tone generation is openly available.^6^ The stimuli consisted of a 10 kHz pure tone applied for 30 s and modulated at an amplitude of 53.71 Hz sine wave (100% modulation depth) and set to 85 dB SPL (Brüel & Kjaer type 2238 sound level meter).

The timestamps locked to the peaks and valleys of the modulated tone were recorded through the Open Ephys digital input port. Via a linear interpolation, these time values were used to obtain an instantaneous phase time series, which in turn was used to reconstruct the signal. This allowed the time-frequency analysis to remain engaged to the auditory stimulation (Amaral-Júnior et al., 2019).

Finally, to track the animal’s movement, the whole experiment was filmed (640 × 480 resolution; 30 frames per second) by a camera (Logitech^®^, C270 Hd 720p) set at the top of the arena.

### Data Analysis

The data were analyzed off-line using custom-made codes and standard MATLAB functions (MATLAB R2020a Signal Processing Toolbox and EEGlab toolbox).^7^ For the analysis of LFP, the signal was decimated to 3 kHz. Time-frequency power and the power spectrum density of the evoked potentials were analyzed by using the standard spectrogram (non-overlapping, 16,384-point, Hamming window) and pwelch (non-overlapping, 4,096-point, Hamming window) Matlab functions, respectively. For the analysis of neuronal units, the signal was processed in an unsupervised algorithm for spike detection and sorting (Chaure et al., 2018).

The open-source software Bonsai (Lopes et al., 2015) was used to track the animal over the arena. From the x and y coordinates of each video frame, we created a tracking map and calculated the displacement and the total distance traveled.

## Results

### Local Field Potentials

Long-lasting modulated tones can evoke a stable oscillatory activity at different levels of the auditory system (Picton et al., 2003). More specifically, the IC can be entrained by a programmed frequency modulation (Lockmann et al., 2017; Simões et al., 2020).

**FIGURE 6:**
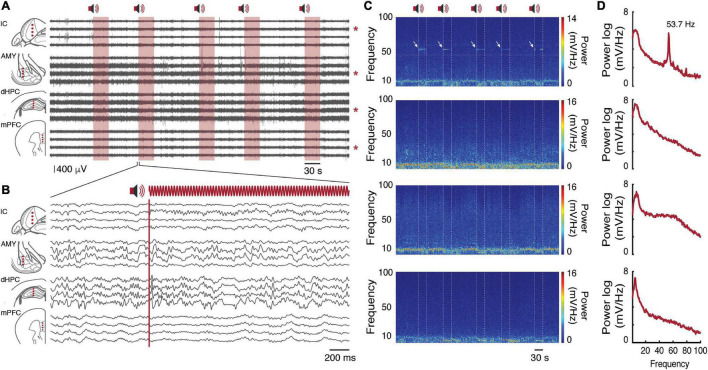
Local Field Potentials. **(A)** Raw record of sixteen channels on each brain substrate. In red we have the auditory stimulus presentation window. Pictorial representations of channels positioned in each brain region are represented on the left. Each red asterisk on the right represents the channel chosen for the time-frequency analysis. **(B)** Two-second time window of the second auditory stimulus presentation. Filtered records between 1 and 100 Hz. **(C)** Channel time-frequency power spectrogram signaled by red asterisks in **(A)**. From top to bottom: IC, AMY, dHPC, mPFC. **(D)** Channel mean power spectral density of the evoked potentials signaled by red asterisks in **(A)**. From top to bottom: IC, AMY, dHPC, mPFC.

As expected, over five stimulus presentations, the oscillatory activity in the IC showed a distinct power spectral signature at the same modulated frequency (53.7 Hz) Figure 6C—Top and Supplementary Figure 2). Moreover, the mean power value at the programmed frequency modulation was quiet representative in the frequency spectrum (Figure 6D—Top). It is worth mentioning that, according to the IC tonotopic organization, dorsal and central IC perfectly responds to mid frequencies (10 kHz) (Clopton and Winfield, 1973; Stiebler and Ehret, 1985; Malmierca et al., 2008).

As described above, Mouse 1 had four electrode arrays distributed over the IC, AMY, dHPC, and mPFC. Each electrode array had four tungsten wires in four dorsal-ventral coordinates (Figure 2F). According to the records, each brain region showed a distinct oscillatory pattern, without visible crosstalk contamination between channels (Figures 6A–C). While the largest amplitude theta oscillations were observed at the dHPC (Buzsáki, 2002), theta and other rhythms can also be observed at both substrates with different features (Soltani Zangbar et al., 2020; Cohen et al., 2021).

### Single Units

**FIGURE 7:**
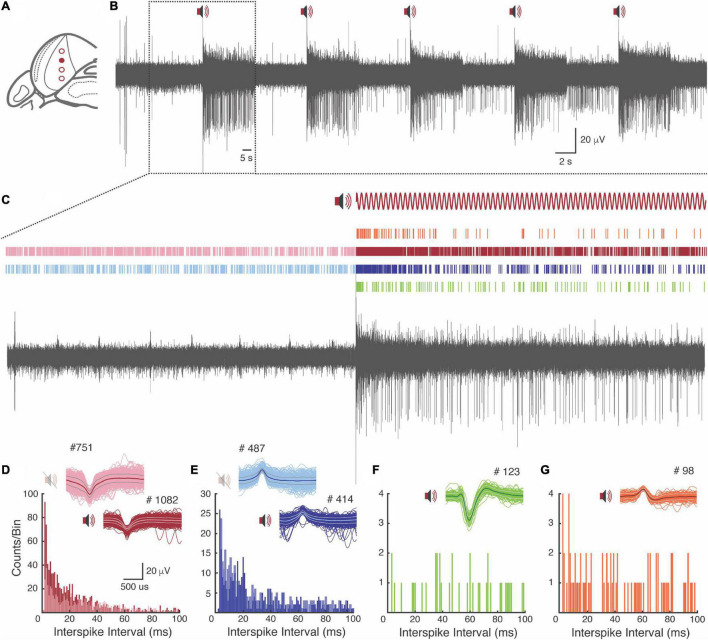
Single units sorting. **(A)** Pictorial representation of channels positioned in the IC. The dot filled in red represents the channel chosen for the analysis. **(B)** Full record filtered between 300 and 3,000 Hz with the respective auditory stimuli presentations. **(C)** Sixty-second time window, before and during the auditory stimulus presentation. Above the record is represented four types of cells and their respective firing rate over time. **(D–G)** Interspike-interval histogram of each cell type. Above the histograms, the respective waveform and the total firing rate (#) are represented.

We identified four cell types in the ventral IC that substantially changed the activity to the auditory stimuli (Figure 7). Interestingly, two cells exhibit a continuous firing, but while one increases the firing rate in front of the stimulus (Figures 7C,D—red cell), the other one keeps the firing rate constant, changing its timing according to the modulation frequency period (Figures 7C,E—blue cell). The other two cells respond only in front of the stimulus (Figures 7C,F—green cell; Figures 7C,G—orange cell).

Prominent single units can also be qualitatively observed along the sixteen recorded channels (Supplementary Figure 1).

### Exploratory Behavior

**FIGURE 8:**
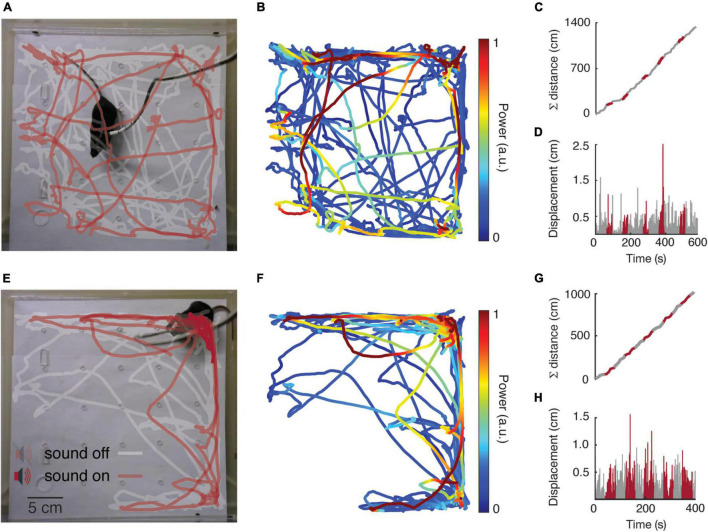
Exploratory behavior analysis. Mouse 1 and Mouse 2 are represented at the top and bottom of the figure, respectively. **(A,E)** Tracking maps. **(B,F)** Ventral IC power spectral at the same modulated frequency band (53.7 ± 0.3 Hz) on the tracking map. **(C,G)** Accumulated distance over time. **(D,H)** Displacement over time.

The exploratory behavior was analyzed in order to identify any impairment of the animal’s locomotion, in face of surgery and/or the new headstage design. However, in both animals, the accumulated distance and the displacement over time showed a linear and constant distribution (Figure 8), with distances covered by more than 10 m at the end of the protocol. In general, during the experimental protocol, the animals did not show signs associated with pain, discomfort and/or distress (Supplementary Video 3).

## Discussion

Over the past few years, open-source initiatives have encouraged the development of investigation methods that are accessible to research groups. Some scientific teams have developed and shared cutting-edge methodologies (Lopes et al., 2015; Mathis et al., 2018) and equipment (Siegle et al., 2017) free of charge or made available at nominal cost. An important step to ensure reproducibility and accuracy with a high degree of agreement is not to rely on ordinary equipment in which almost no knowledge of the internal workings is released to the end-users.

Based on this democratic initiative of accessible and equitable science, which involves developing more affordable materials and methods, we designed an adaptation to the RHD headstage system (Intan technologies), associated with a flat connector (SMD/FPC) to replace the traditional Omnetics connector. It is unquestionably that the Omnetics connectors have a high technological level and are widely used by the scientific community in several applications. However, these may lead to prohibitive costs for underfunded research groups (especially in developing countries) and are a limiting factor when designing experimental protocols. On the other hand, the flat connector has affordable costs, is easily recycled, and can be also easily adapted to the experiments. Furthermore, the new connector proposed in its flat format can avoid damage from repetitive use, since the connection has no pins and does not need a certain effort to fit. Its connection can be made only by a smooth positioning inside the flat connector and then affixed by a small black lock that strongly holds the Flat-Grid Connector (Figure 1B). More information can be found in Supplementary Material.

For proper connection with the new connector, we designed a small circuit board with a through holes grid, where the electrodes are soldered. Besides that, a complete structure for the placement of the electrode arrays was designed to adapt to the specific needs of different projects.

It is noteworthy that the total weight of the headstage and the implanted device is around 2 g, therefore the experimental animals easily adapted to the headstage design, without discomfort or impairments not interfering in its mobility and allowing the task execution. Besides, the extracellular records had great quality with detectable evoked responses to an auditory task both on local field and unit activities.

As already described, there is a wide range of commercial electrodes for recording electrophysiological activity. These electrodes with their different shapes and arrangements make use of different materials and are mostly accompanied by omnetics connectors (Buzsáki et al., 2015; Jun et al., 2017; Liu et al., 2021). We suggest that a way to pair these high-tech electrode arrays in our adapted recording headstage would be the development of simple adapters. Adapters developed in the same way that we suggested in Supplementary Material to couple the Flat-Grid Connector to the commercial headstage of Intan Technologies (see text footnote 3). Small changes in the available circuit could bring new possibilities for carrying out experiments with different equipment.

It is reasonable to assume that versatile and flexible tools allow the study of biological processes that are not observable with ordinary equipment. Moreover, the development of customized equipment with affordable costs can enable local scientific development, as it inspires scientific endeavor among students, teachers, and supervisors alike. Overall, compared to the commercial versions in which the total cost is around 700 to 900 dollars (RHD Headstages—Model: RHD 2216 or RHD 2132; Intan Technologies), for only the recording headstage, or even more than 1,000 dollars (Low-profile SPI Headstage 64ch; Open-Ephys.org), in contrast our solution cost around 350 to 450 dollars taking into consideration the full set, composed of the headstage and the electrode arrays. Our project is available through open-source initiatives (see text footnote 3) and may be modified by collaborators when adding new features to both developed segments.

## Data Availability Statement

The raw data supporting the conclusions of this article will be made available by the authors, without undue reservation.

## Ethics Statement

The animal study was reviewed and approved by Institutional Animal Care and Use Committee at the Universidade Federal de Minas Gerais (CEUA-UFMG: 198/2019).

## Author Contributions

FM, LG, PA, and MM conceived and designed the projected. FM, LG, PA, VC, and MM designed boards and connectors. FM performed surgery and electrophysiological records, electrophysiological and behavioral analysis, prepared figures, and drafted the manuscript. FM and LG performed behavioral tests. EM supervised and contributed to refinements of the system. All authors revised and approved the final version of the manuscript.

## Conflict of Interest

The authors declare that the research was conducted in the absence of any commercial or financial relationships that could be construed as a potential conflict of interest.

## Publisher’s Note

All claims expressed in this article are solely those of the authors and do not necessarily represent those of their affiliated organizations, or those of the publisher, the editors and the reviewers. Any product that may be evaluated in this article, or claim that may be made by its manufacturer, is not guaranteed or endorsed by the publisher.

## Footnotes

1
https://cutt.ly/UcSeYPd

2
Open-Ephys.org

3
https://github.com/fgmourao/Lab_Projects

4
https://cutt.ly/ecAM15O

5
https://cutt.ly/QcrUCvL

6
https://cutt.ly/FzHgv7H

7
https://sccn.ucsd.edu/eeglab/index.php
