# Supplementary material for: A Fully Adapted Headstage With Custom Electrode Arrays Designed for Electrophysiological Experiments
Source: Front Neurosci. 2022 Mar 3;15:691788. doi: 10.3389/fnins.2021.691788 (PMC8928121; doi:10.3389/fnins.2021.691788)

A Fully Adapted Headstage With Custom Electrode Arrays Designed for Electrophysiological Experiments

Supplementary Material – Tutorials

Authors: Flávio Afonso Gonçalves Mourão[a], Leonardo de Oliveira Guarnieri [ab], Paulo Aparecido Amaral Júnior [ab], Vinícius Rezende Carvalho a[b], Eduardo Mazoni Andrade Marçal Mendes [ab], Márcio Flávio Dutra Moraes [a].

a. Núcleo de Neurociências, Departamento de Fisiologia e Biofísica, Instituto de Ciências Biológicas (ICB), Universidade Federal de Minas Gerais (UFMG). Av. Antônio Carlos, 6627 - CEP 31270-901. Belo Horizonte, Minas Gerais, Brazil.

b. Programa de Pós-Graduação em Engenharia Elétrica, Departamento de Engenharia Eletrônica (DELT), Escola de Engenharia, Universidade Federal de Minas Gerais (UFMG). Av. Antônio Carlos, 6627 - CEP 31270-901. Belo Horizonte, Minas Gerais, Brazil.

Correspondence:

Flávio Afonso Gonçalves Mourão, PhD
Email: fagm@ufmg.br

Márcio Flávio Dutra Moraes, PhD
Email: mfdm@icb.ufmg.br

2021

Recording Headstage

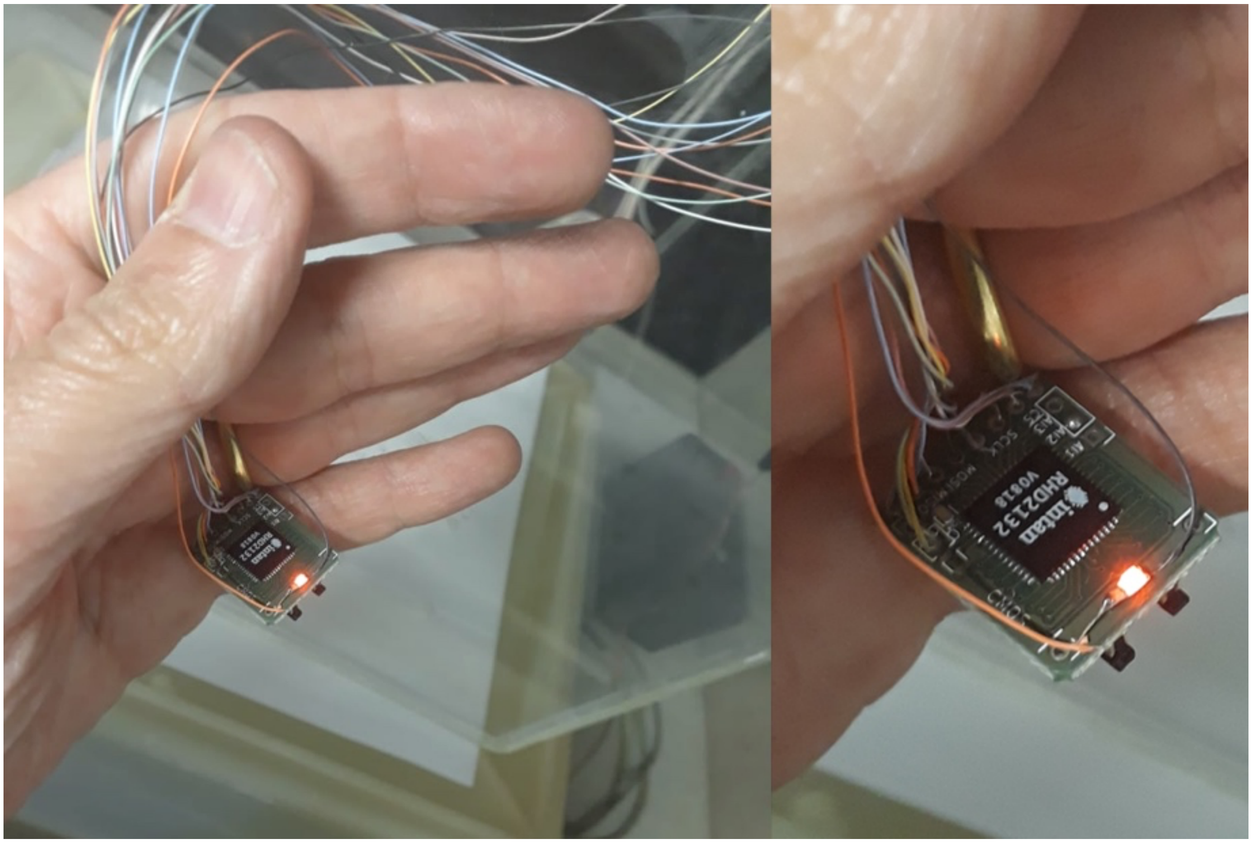

** Led is not in the original project*

Component	Specification	Supplier	Price	Quantity	Link
FPC connector (Flexible Printed Circuits)	SMD vertical type 0.5mm pitch 2mm height	mktechnic	$0.79(un)	2	https://cutt.ly/Xx55qQE
RHD2132 amplifier chip	32-channel unipolar inputs and common reference	Intan Technologies	$390 (un)	1	https://cutt.ly/Yx6qief
Resistor	SMD – 0 Ω	-	-	1	-
Capacitor	SMD – 10 nF	-	-	1	-
Capacitor	SMD – 100 nF	-	-	2	-

Components

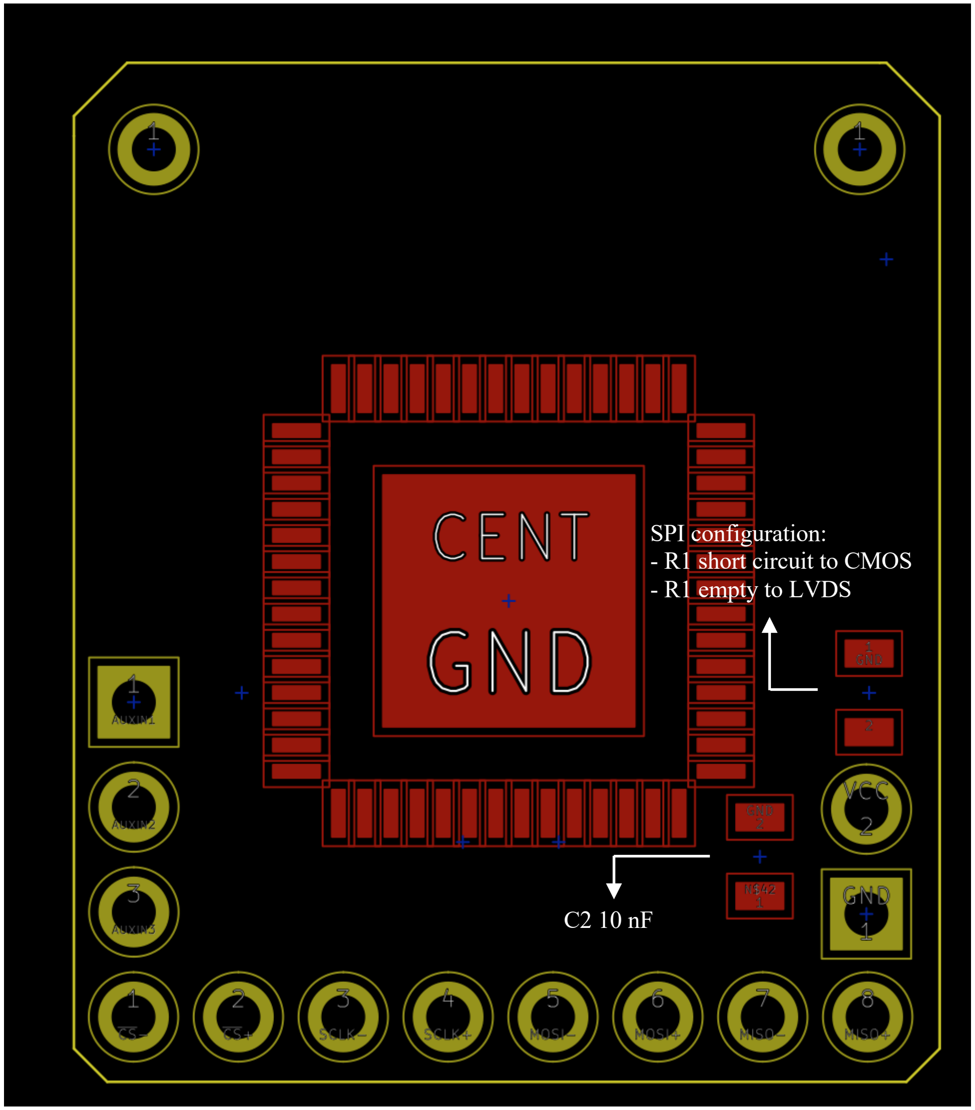
CENT
GND
SPI configuration:
- R1 short circuit to CMOS
- R1 empty to LVDS
C2 10 nF

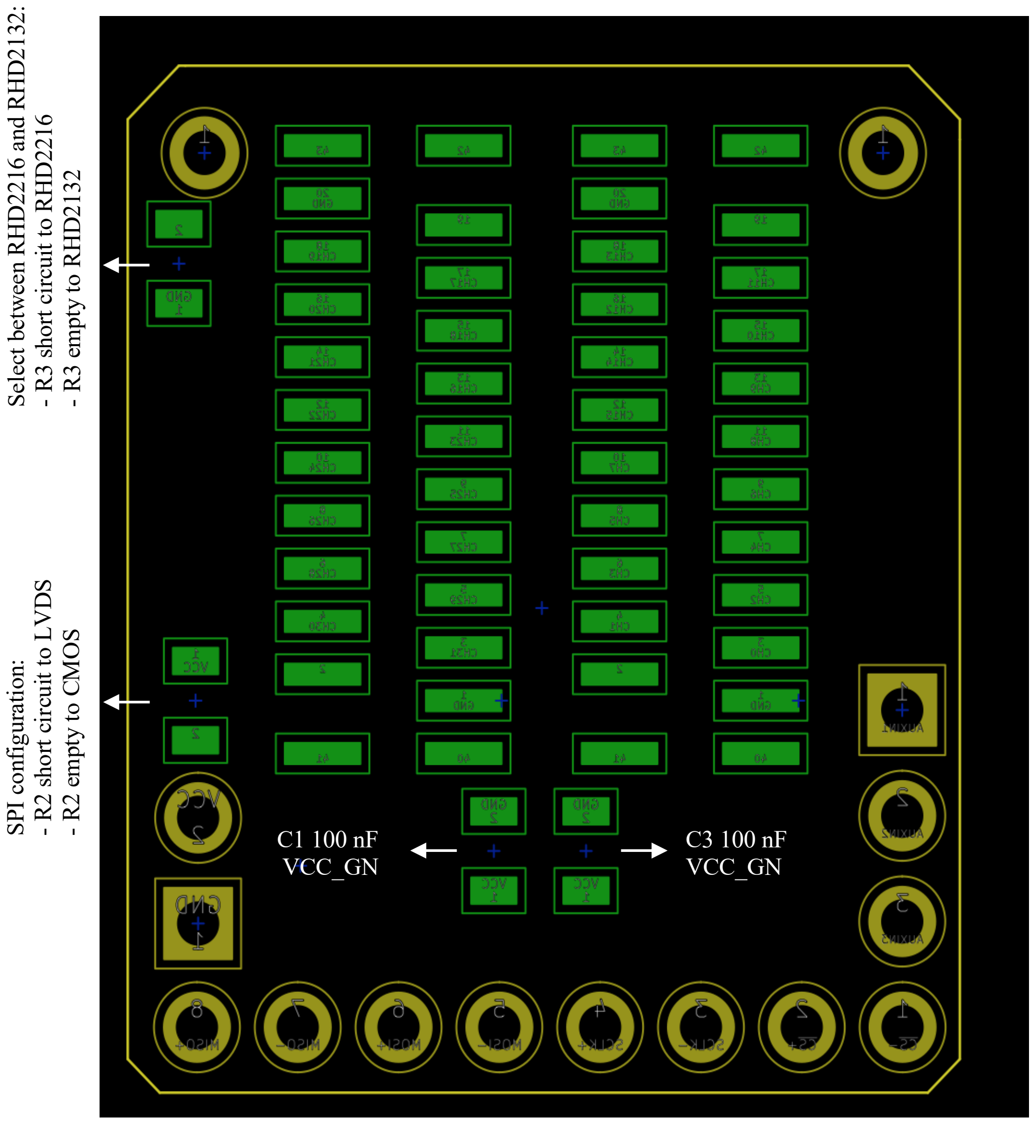
Select between RHD2216 and RHD2132:
- R3 short circuit to RHD2216
- R3 empty to RHD2132
SPI configuration:
- R2 short circuit to LVDS
- R2 empty to CMOS
C1 100 nF
VCC_GN
C3 100 nF
VCC_GN

Handmade cable

We strongly recommend the following tutorial:

- https://open-ephys.atlassian.net/wiki/spaces/OEW/pages/491587/Fine+wire+tether

Component	Specification	Supplier	Price	Quantity	Link
Omnetics Polarized PZN	Straight Thru-Hole (Type DD) 12 contacts	Omnetics Connector Corporation	*on request*	1	https://cutt.ly/FcqiCNQ
Micro bare copper wire	CZ 1187 wire series	Cooner Wire	*on request*	12	https://cutt.ly/lcqo9Pz

However, in our adapted board, each contact needs to be checked

Wiring diagram:

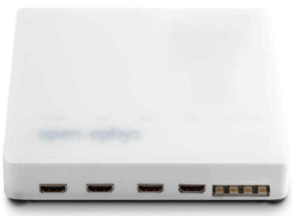

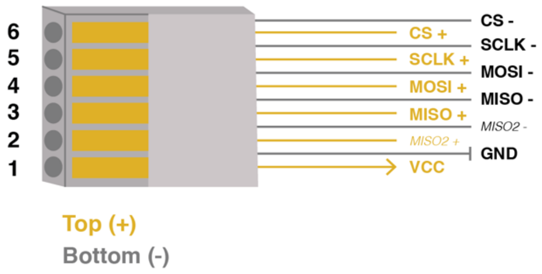

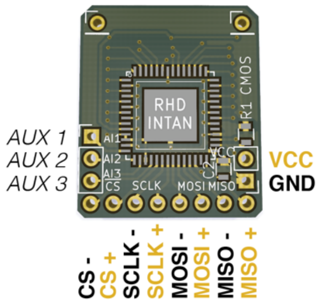

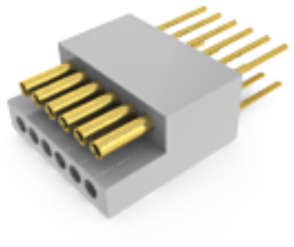

Flat-Grid Connector – V2 – Current Version

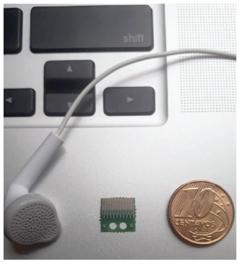

Channels Map

Be careful with the board position

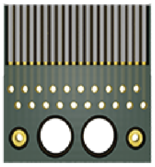

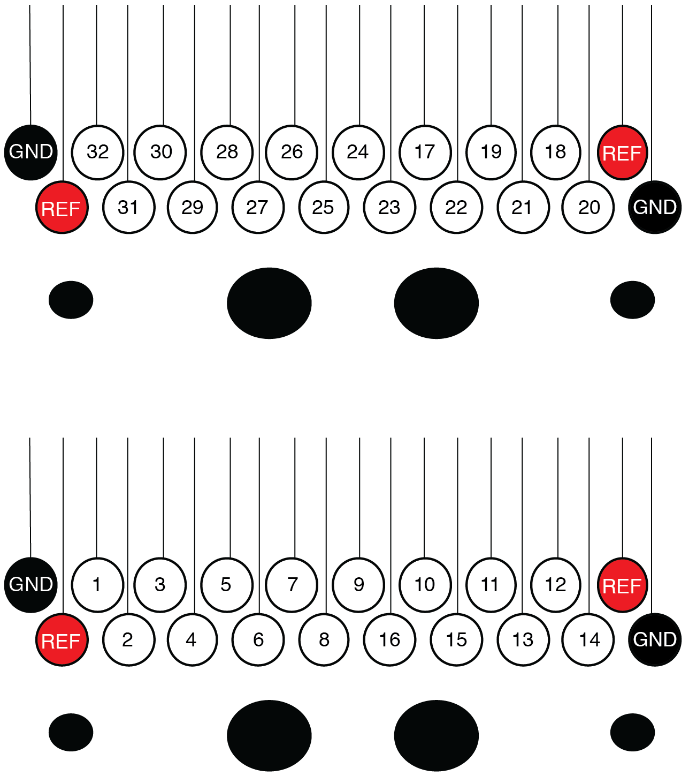

Bottom view

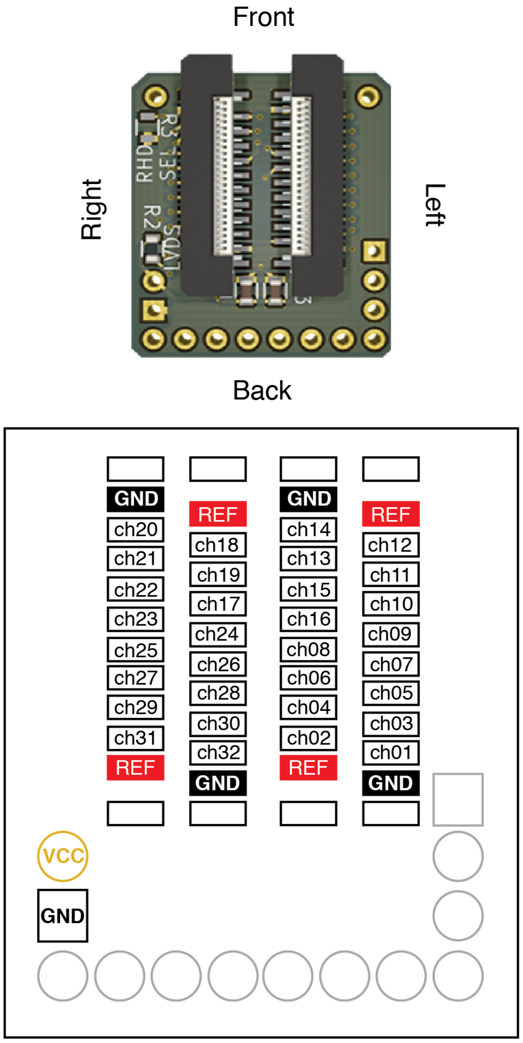

Component	Specification	Supplier	Price	Quantity	Link
Stainless steel wire	0.127 mm internal diameter, teflon-coated. 100 feet. Model 7914	A-M Systems Inc	$140 (un)	1	https://cutt.ly/5x6eIoR
Tungsten microwires					
Silver paint	1 troy oz. (31.1g)	SPI Supplies®	$50 (un)	1	https://cutt.ly/Ox6sup1
Thermosetting polymer	5 minute Epoxy	Devcon	-		https://cutt.ly/Zx6ltrE<br>https://cutt.ly/mx6zobJ<br>https://cutt.ly/Rx6zFZZ

Flat-Grid Connector – V1 – Old Version

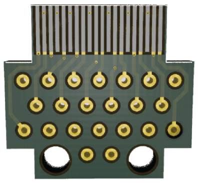

Channels Map

Be careful with the board position

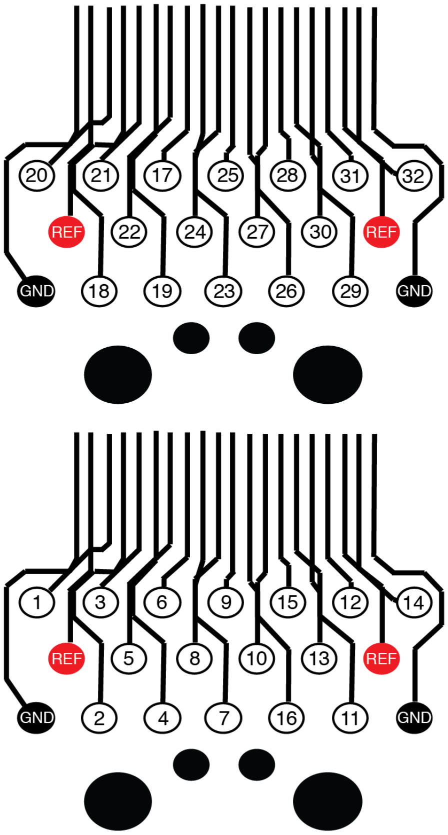

Bottom view

Front

Right

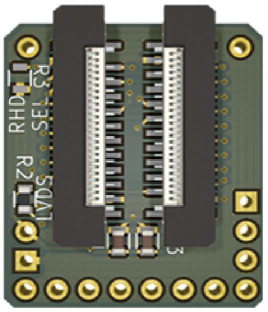

Left

Back

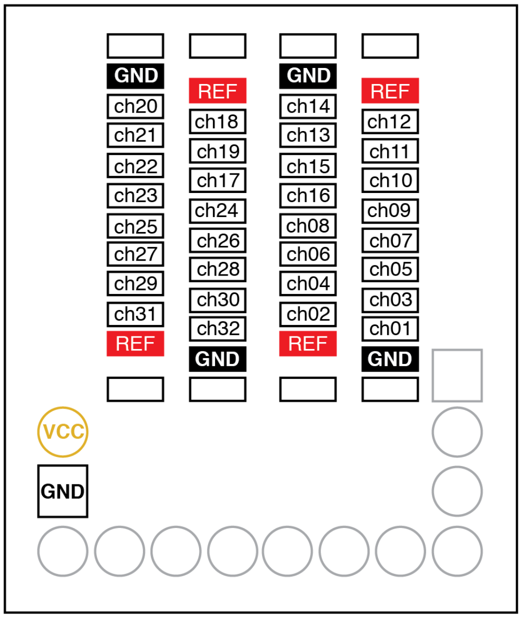

SMD/FPC connector ← → Omnetics
Adapters for the original RHD headstage

Component	Specification	Supplier	Price	Quantity	Link
FPC connector (Flexible Printed Circuits)	SMD vertical type 0.5mm pitch 2mm height	mktechnic	$0.79(un)	2	https://cutt.ly/Xx55qQE
Omnetics Neuro Nano Strip	A79042-001 NPD-18-VV-GS	Omnetics Connector Corporation	*on request*	1	https://cutt.ly/UcqDO66
Omnetics Neuro Nano Strip	A79046-001 NPD-36-DD-GS	Omnetics Connector Corporation	*on request*	1	https://cutt.ly/UcqDO66

RHD 16-Channel Recording Headstages

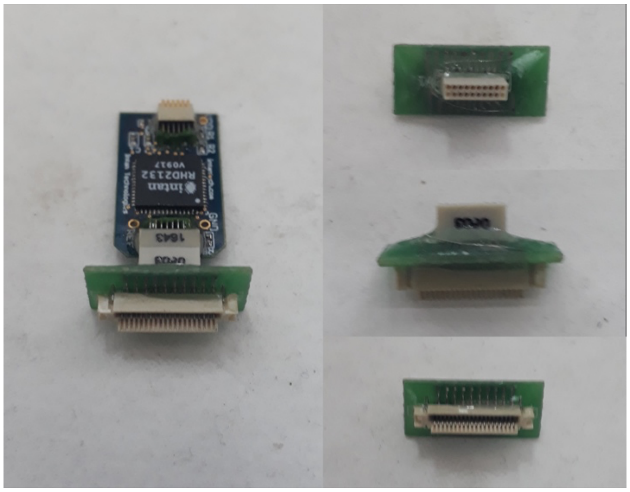

RHD 32-Channel Recording Headstages

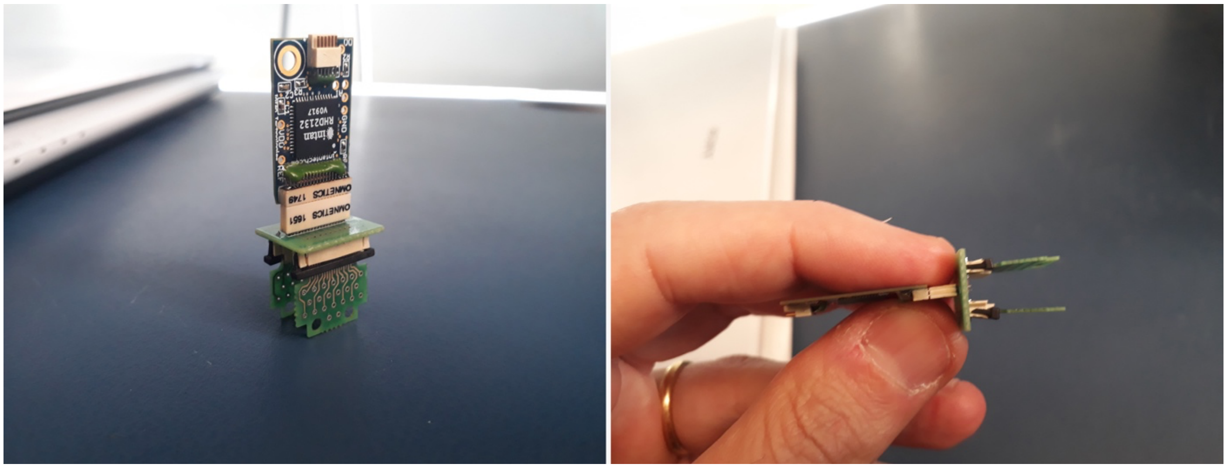

Supplement: Supplementary file 1 [file Data_Sheet_1.pdf]
